# Phylogenetic Characterization and Pathogenicity in Cattle and Pigs of Foot-and-Mouth Disease Viruses Circulating in Myanmar Between 2016 and 2022

**DOI:** 10.1155/tbed/1532487

**Published:** 2025-10-29

**Authors:** Rie Kawaguchi, Tatsuya Nishi, Katsuhiko Fukai, Khin Ohnmar Lwin, Kazuki Morioka

**Affiliations:** ^1^Transboundary Animal Disease Research Division, National Institute of Animal Health, National Agriculture and Food Research Organization, 6-20-1, Josui-honcho, Kodaira, Tokyo 187-0022, Japan; ^2^Veterinary Medicine and Disease Control Division, Livestock Breeding and Veterinary Department, Ministry of Agriculture, Livestock and Irrigation, Office Building No. (36), Pantita Street, Zeyar Thiri Township, Nay Pyi Taw 15013, Myanmar

## Abstract

Foot-and-mouth disease (FMD) is a highly contagious and serious transboundary disease affecting cloven-hoofed animals. Myanmar is a critical area for FMD outbreaks in Southeast and East Asian regions because of its geographical location bordering South Asian countries and its cattle industry. Phylogenetic characterization and pathogenicity in susceptible animals of circulating viruses in Myanmar are essential to prepare the rapid and accurate diagnosis and implement effective FMD prevention. This study analyzed a total of 34 vesicular epithelial samples collected from FMD cases in northern, central, and southern Myanmar between 2016 and 2022. Phylogenetic analysis of VP1 nucleotide sequences revealed multiple serotypes and topotypes between 2016 and 2019, including serotype O/Middle East-South Asia (ME-SA) topotype (O/ME-SA/Ind-2001e) and Southeast Asia (SEA) topotype, and serotype A/ASIA topotype. Subsequently, all viruses across Myanmar detected from 2019 to 2022 belonged to O/ME-SA/Ind-2001e. Phylogenetic analysis of the whole genome sequence showed that O/ME-SA/Ind-2001e viruses detected after 2019 were classified into a different genetic group with those of 2016 isolates in Myanmar. Based on phylogenetic analysis, one representative strain from 2019 that was genetically similar to viruses detected from 2019 to 2022 and to a 2022 Indonesian isolate was selected for pathogenicity testing in comparison with a 2016 strain closely related to viruses from neighboring countries. Both strains were used for experimental infection in pigs and showed similar pathogenicity. The 2019 strain was additionally tested in cattle and caused typical FMD pathogenicity, including vesicular development and virus excretion. Viral genes and antibodies in infected animals were detectable using existing diagnostic methods, which are considered useful for identifying currently circulating viruses. These results elucidate the subtypes of FMD viruses (FMDVs) circulating in Myanmar, their phylogenetic relationships with viruses from neighboring Asian countries, their pathogenicity, and the applicability of available diagnostic methods. It offers insights into appropriate control strategies against FMD in Southeast and East Asian regions.

## 1. Introduction

Foot-and-mouth disease (FMD) is a highly contagious and transboundary disease affecting cloven-hoofed animals. Animals infected with FMD typically exhibit vesicular lesions localized to oral and nasal regions, mammary glands, and feet. These infected animals act as a secondary source for viral transmission to susceptible herds [1]. FMD outbreaks cause economic loss due to reduction of livestock productivity and implementation of international commerce restrictions on animals and derivative products [2, 3].

FMD virus (FMDV) is responsible for FMD, belonging to the genus *Aphthovirus* in the family *Picornaviridae* [1]. Its genome is a single-stranded positive-sense RNA approximately 8.4 kb in length and contains with a 7.0 kb open reading frame (ORF) encoding four structural and 10 nonstructural proteins [4, 5]. FMDV consists of immunologically distinct seven serotypes: A, O, C, Asia1, and Southern African Territories (SAT) 1, 2, and 3 [6]. These serotypes are classified into various genetic and geographical subtypes, referred to as “topotypes” or “lineages,” based on the nucleotide sequence similarities of the VP1 region [7, 8]. FMDV circulates in geographically distinct groups known as “pools” categorized from pool 1 to 7. Each pool is composed of unique sets of genetically and geographically distinct viruses with their serotypes and topotypes [9]. In pool 1, frequent outbreaks of serotypes O and A have been reported, along with sporadic outbreaks of serotype Asia 1 [2, 10]. Serotype O is prevalent with various topotypes, including the Middle East-South Asia (ME-SA), Southeast Asia (SEA), and CATHAY topotypes [10, 11].

Myanmar is endemic for FMD and a critical area for disease control. Its geographical location shares borders with India and Bangladesh in South Asia within pool 2, and this has led to trans-pool movements from South Asia to Myanmar in the past, including the serotypes O and Asia 1 [12–14]. Additionally, Myanmar has the largest population of cattle and buffaloes in SEA [15] and there is information on the formal and informal trade of live animals with neighboring countries, such as Thailand, Vietnam, Cambodia, the Lao People's Democratic Republic, and China [15, 16]. The FMD outbreaks in Myanmar can trigger outbreaks in neighboring countries due to livestock movement. Viruses isolated from previous outbreaks in East Asian countries have been reported to belong to the same genetic lineage as those circulating in Myanmar during the same period [17]. Therefore, phylogenetic characterization and pathogenicity in susceptible animals of circulating viruses in Myanmar are essential to prepare the rapid and accurate diagnosis and implement effective prevention of FMD in Southeast and East Asian regions. In this study, genome sequences were collected from field cases between 2016 and 2022 in Myanmar, and the pathogenicity of the isolated strains in cattle and pigs was elucidated through experimental infections.

## 2. Materials and Methods

### 2.1. Sample Collection, Virus Isolation, and RNA Extractions From Epithelial Samples

**Figure 1:**
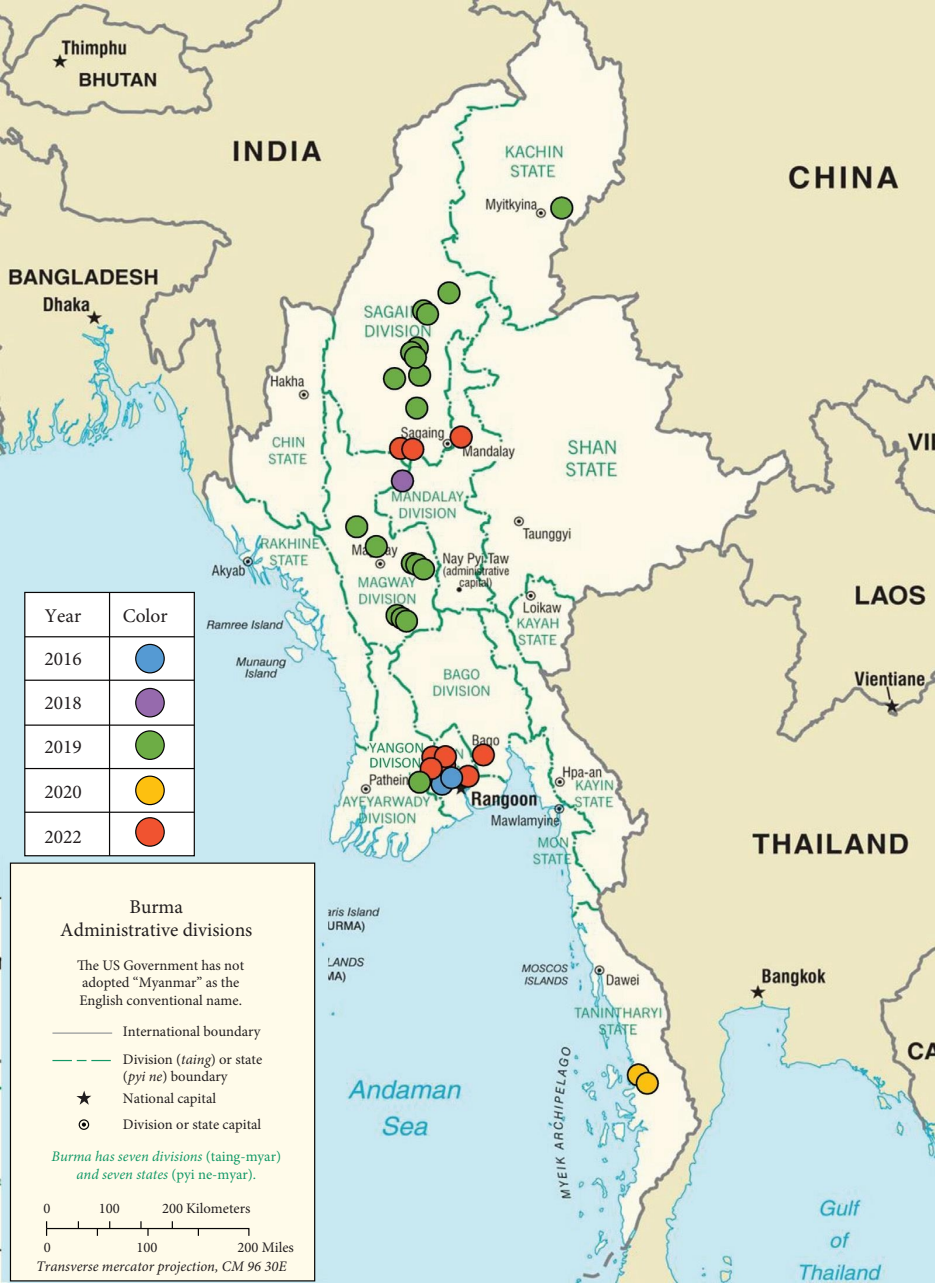
Geographical and temporal distributions of confirmed FMDV outbreaks during 2016–2022. The map was sourced from the CIA World Factbook (https://www.cia.gov/the-world-factbook/maps/). Dotted blots represent each virus sample and are colored according to the year of the outbreak, as shown in this figure.

Epithelial samples were collected from cattle and pigs with clinical FMD symptoms in Myanmar between 2016 and 2022. Their geographical and temporal distributions are mapped using the CIA World Factbook (https://www.cia.gov/the-world-factbook/maps/) (Figure 1). Samples were initially transported to the FMDV Diagnostic Laboratory in Myanmar and then to the Kodaira Research Station, National Institute of Animal Health (NIAH) in Japan, for further analysis.

Epithelial samples were homogenized in a small volume of Dulbecco's Modified Eagle Medium/Nutrient Mixture F12 (DMEM; Thermo Fisher Scientific, Waltham, MA, USA) and centrifuged at 4 °C at 8000 × *g* for 10 min. The resulting solutions were inoculated into monolayers of ZZ-R 127 cells [18] and incubated at 37°C for 1 h. The solution from each flask was then discarded and washed with DMEM. Next, the DMEM supplemented with 10% fetal bovine serum (FUJIFILM Wako Chemicals Corporation, Osaka, Japan) was added to each flask, and the flasks were incubated at 37°C until cytopathic effects (CPEs) were detected. After CPE was observed, the presence and serotype of the isolated viruses were confirmed using an FMDV lateral flow antigen detection system with monoclonal antibodies reactive with multiple FMDV serotypes [19]. Viral RNAs were extracted from the sample solutions or viral isolates isolated from the cell cultures using a High Pure Viral RNA kit (Roche Diagnostics, Basel, Switzerland).

### 2.2. Reverse Transcription-Polymerase Chain Reaction (RT-PCR) and Sequencing of VP1 Coding Region

Extracted RNAs were reverse transcribed and amplified using RT-PCR with a SuperScript IV One-Step RT-PCR System (Invitrogen, Waltham, MA, USA) to target the VP1 coding region of the FMDVs with the following primers: O-1C272F (5′-TBGCRGGNCTYGCCCAGTACTAC-3′), O-1C283F (5′-GCCCAGTACTACACACAGTACAG-3′), A-1C562R (5′-TACCAAATTACACACGGGAA-3′), and EUR-2B52R (5′-GACATGTCCTCCTGCATCTGGTTGAT-3′) [20]. RT-PCR was performed under the following conditions: 55°C for 10 min and 98°C for 2 min, followed by 35 cycles (98 °C for 10 s, 55°C for 10 s, and 72°C for 30 s) and then 72°C for 5 min. PCR products were purified using a QIA quick PCR purification Kit (Qiagen, Hilden, Germany), sequenced using a Big Dye Terminator v3.1 Cycle Sequencing Kit (Thermo Fisher Scientific) with the following primers: O-CRH2F (5′-GAYTACGCSTACACSGCGTC-3′), A-1C612F (5′-TAGCGCCGGCAAAGACTTTGA-3′), and NK72 (5′-GAAGGGCCCAGGGTTGGACTC-3′) [20]. Sequencing was performed under the following conditions; 96°C for 1 min, followed by 25 cycles (96°C for 10 s, 50°C for 5 s, and 60°C for 4 min). Sequencing products were purified using Clean Seq (Beckman coulter, Indianapolis, IN, USA), and nucleotide sequences of the purified products were determined using a 3500 Genetic Analyzer (Thermo Fisher Scientific).

### 2.3. Whole Genome Sequencing

Extracted RNAs from the isolated viruses and epithelial samples from 2020 to 2022 were processed for whole genome sequencing analysis. Fragments of the viral whole genome were amplified using a SuperScript IV One-Step RT-PCR System and the primer sets as follows: Set 1 consisted of 5′NT-Fa (5′-CCGTCGTTCCCGACGTTAAAGGG-3′) and 2B331R (5′-GGCACGTGAAAGAGACTGGAGAG-3′), and set 2 consisted of 2B217F (5′-ATGGCCGCTGTAGCAGCACGGTC-3′) and 3′NT-Ra (5′-RCTTTTCACYCCTRYGGYGTC-3′), 3′NT-Rb (5′-AAGCCYTTTYRGRCTTTTCACYCC-3′) or 3′NT-Rc (5′-CGCCTCAGAGTCTTTCTGCCAATTG-3′) [21]. PCR products were purified using the QIA Quick PCR Purification Kit. The libraries were constructed using the Ion Xpress Plus fragment library kit (Life Technologies) through fragmenting DNA for 8 min to produce an average fragment size of 300 bp. Barcoding was performed using Ion Xpress barcodes (Life Technologies). Libraries were then size selected using 2% E-Gel SizeSelect (Invitrogen). The fragment size and concentration were assessed using an Agilent Bioanalyzer. Amplification of libraries was performed using the Ion OneTouch 2 system using the 314 chip (version 2) with an Ion PGM Template OT2 200 kit (Life Technologies) and sequenced using an Ion Torrent PGM sequencer and Ion PGM Sequencing 200 kit (version 2) (Life Technologies). Sequence assembly and mapping were conducted using Torrent Suite software version 5, with default parameters and a threshold coverage of 15.

### 2.4. Phylogenetic Analysis

Alignment and phylogenetic study of the VP1 and ORF nucleotide sequences for serotype O were analyzed using MEGA software (version 11.0; http://www.megasoftware.net/). VP1 sequences were compared with prototype strains provided by the World Reference Laboratory for FMD (WRLFMD), and field isolates selected based on over 92% of identity rates by a BLAST search for each country and outbreak year. ORF sequences of the Ind-2001e lineage within the ME-SA topotype (ME-SA/Ind-2001e) of serotype O were compared with the ME-SA/Ind-2001e isolates based on over 95% of identity rates by a BLAST search. Sequences were aligned using ClustalW, and phylogenetics were analyzed using the maximum-likelihood method with the Kimura [22] 2-parameter model.

### 2.5. Experimental Infections With Field Isolates From Myanmar

In this study, field strains isolated from outbreaks in Myanmar, O/MYA/Yan/5/2016 [23] and O/MYA/Mgy/11/2019, classified within the ME-SA/Ind-2001e, were utilized for experimental infections to elucidate their pathogenicity in susceptible animals (Supporting Information 4: Table S1). These two isolates were initially isolated using ZZ-R 127 cell and subsequently propagated on LFPK-α_v_β_6_ cells [24].

In Experiment 1, the pathogenicity of the two strains was clarified in pigs via the intraoral route, representing the natural infection pathway observed under field conditions, using methods published by Fukai [25]. Two 2-month-old pigs were inoculated with O/MYA/Yan/5/2016 (Nos. 194 and 195) or O/MYA/Mgy/11/2019 (Nos. 2113 and 2114), respectively, with 1 mL of 10^6.0^ of 50% tissue culture infectious dose (TCID_50_) via the intraoral route.

In Experiment 2, the pathogenicity of O/MYA/Mgy/11/2019 was clarified in cattle and pigs via the intradermal route, which is the most established route for FMDV experimental infections. Two 6-month-old cattle (Nos. 211 and 233) and four 2-month-old pigs (Nos. 213, 214, 2111, and 2112) were inoculated with 1 mL of 10^6.0^ TCID_50_ via the intradermal route on their tongue (cattle) or right and front heel bulbs (pigs). Pathogenicity in susceptible animals was compared with previous studies on experimental infections with FMDV serotype O (O/JPN/2010) using the same methods [26].

Following virus inoculation, blood, oral, and nasal swabs were acquired to 8–10 days postinoculation (dpi), following previously described methods [26]. Clinical signs were assessed using a modified version of the clinical scoring system described by Rainwater-Lovett [27], which assigned 1 point each for the presence of vesicular lesions on fore and hind limbs (1 point per limb), snout, lip, and 1 point for fever (40°C or higher), with a maximum daily score of 7 per animal.

### 2.6. Ethics

All animal procedures were approved by the Animal Care and Use Committee of the NIAH approved prior to the experiments (authorization numbers: 20-058, 21-031, 21-059, 23-020). Experimental infections involving live viruses were conducted in cubicles approximately 14 m^2^ in size, maintained at 25°C with 10−15 air changes per hour, within a high-containment facility at the NIAH. This facility adhered to the Group 4 pathogen containment standards outlined in the World Organization for Animal Health (WOAH) Terrestrial Manual (version 2018) [28]. Throughout the experiments, rectal temperatures of the animals and their behavior and physiology were observed daily by veterinarians. At the end of the experimental period, animals were sedated with xylazine and euthanized using propofol (cattle) or ketamine (pigs), and subjected to necropsy.

### 2.7. Virus Titration, FMDV-Specific Gene Detection, and Antibody Detection

Viruses were titrated to quantify the virus in each clinical sample using LFPK-α_v_β_6_ cells. The LFPK-α_v_β_6_ cells were seeded into 96-well plates at density of approximately 10,000 cells per well on the day of virus isolation, using the DMEM supplemented with 10% FBS. Serial dilutions of the clinical samples, 100 µL each, were inoculated into 5 replicate wells per dilution and incubated at 37°C in an atmosphere of 5% CO_2_. After 72 h of culture, cells were observed for CPE using an inverted microscope (CKX53, Evident, Tokyo, Japan). Viral titers were determined using the Reed-Muench method, based on the appearance of CPE [29]. Viral RNA was extracted from clinical samples using a High Pure Viral RNA Kit (Roche Diagnostics) according to manufacturer instructions. FMDV-specific genes were detected in the extracted RNA samples using RT-PCR with FM8/9 primers, which amplify 644 bases in the conserved 3D region of all seven serotypes [30]. The antibody against structural FMDV proteins was detected using a PrioCHECK FMDV Type O Antibody ELISA Kit (Applied Biosystems, Waltham, MA, USA).

## 3. Results

### 3.1. Phylogenetic Analysis of VP1 Coding Region

**Figure 2:**
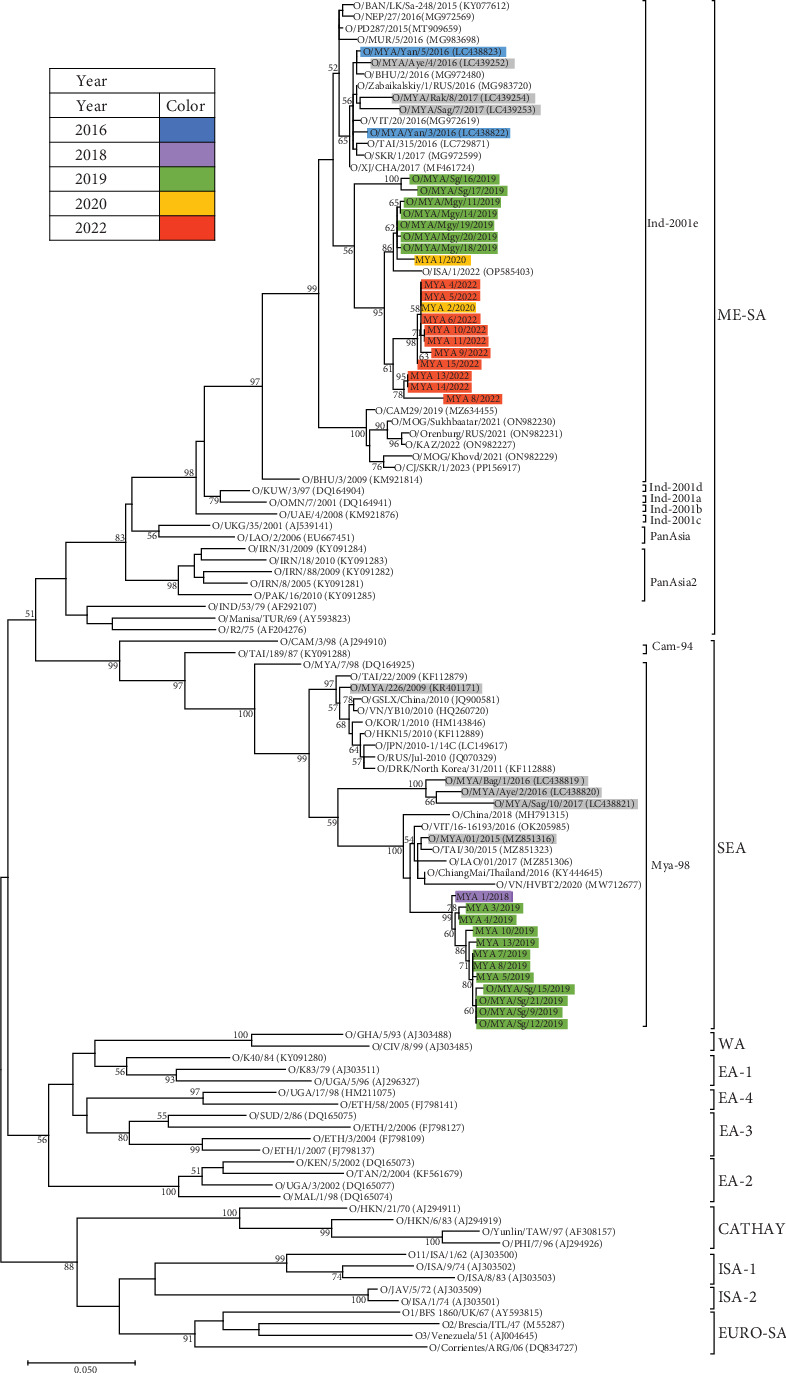
Phylogenetic tree based on VP1 sequences with FMDV serotype O confirmed from outbreaks during 2016–2022. The VP1 nucleotide sequences of FMDV serotype O were phylogenetically analyzed using the maximum likelihood method based on the Kimura 2-parameter model [22]. The horizontal distances are proportional to the minimum number of nucleotide differences required to join the nodes and sequences. The numbers at each node indicate the confidence level of the bootstrap analysis with 500 replicates. Only confidence levels ≥50 are indicated. The isolates or virus samples are colored by year of the outbreak. The grey highlight indicates previously reported VP1 sequences in Myanmar. Reference strains are provided along with the DDBJ/EMBL/GenBank accession numbers in parentheses.

VP1 nucleotide sequences were obtained from RNA extracted from isolated viruses or epithelial samples. Phylogenetic analysis of these VP1 sequences revealed that 19 viral samples were classified as the ME-SA/Ind-2001e, whereas 12 samples were classified as the Mya-98 lineage within the SEA topotype (SEA/Mya-98) of serotype O (Figure 2). Samples collected between 2016 and 2019 belonged to both the ME-SA/Ind-2001e and SEA/Mya-98, whereas all samples collected from 2020 onward were classified within the ME-SA/Ind-2001e lineage. Additionally, one sample from 2019 was classified as the Sea-97 lineage within the ASIA topotype (ASIA/Sea-97) of serotype A (data not shown).

### 3.2. Virus Isolation and Whole Genome Sequences and Phylogenetic Analysis of the ORF Region

Two virus isolates (O/MYA/Yan/3/2016 and O/MYA/Yan/5/2016) were obtained from epithelial samples collected in 2016, along with their whole genome sequences, as reported in the previous study [23]. In the present study, nine viruses were isolated from epithelial samples collected in 2019 (O/MYA/Sg/9/2019, O/MYA/Mgy/11/2019, O/MYA/Sg/12/2019, O/MYA/Sg/15/2019, O/MYA/Sg/16/2019, O/MYA/Sg/17/2019, O/MYA/Mgy/20/2019, O/MYA/Sg/21/2019 and A/MYA/Ygn/24/2019). All virus isolates tested positive with the monoclonal antibodies reactive with multiple FMDV serotypes using the lateral flow device, confirming their identity as FMDV Serotype O and A (data not shown). Whole genome sequences were obtained from nine isolates as well as from 12 epithelial samples collected in 2020 and 2022 without virus isolation. The whole genome sequences were obtained as final assemblies ranging from 7491 to 7691 nucleotides (nt), and the ORF gene, spanning 6999 nt, encoded a polyprotein of 2333 amino acids. All sample information and sequence data, including VP1 sequences, were submitted to the DDBJ database (Table 1).

**Figure 3:**
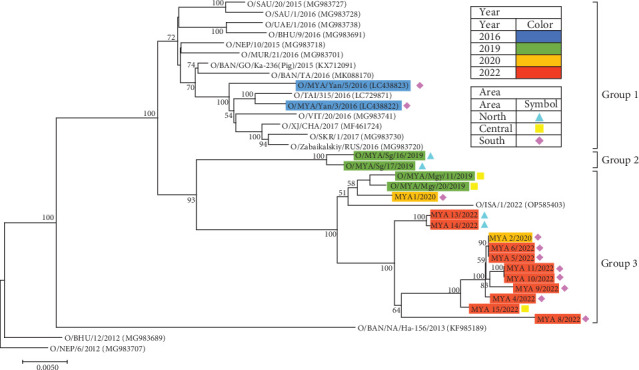
Phylogenic tree of open reading frame (ORF) genes of FMDVs classified as serotype O ME-SA/Ind-2001e. The ORF nucleotide sequences of FMDV ME-SA/Ind-2001e were phylogenetically analyzed using the maximum likelihood method based on the Kimura 2-parameter model [22]. The horizontal distances are proportional to the minimum number of nucleotide differences required to join the nodes and sequences. The numbers at each node indicate the confidence level of the bootstrap analysis with 500 replicates. Only confidence levels ≥50 are indicated. Isolates or virus samples are colored by year and symbolized by area of the outbreak. Reference strains are provided along with the DDBJ/EMBL/GenBank accession numbers in parentheses.

The results of the phylogenetic analysis of the ORF sequences of the ME-SA/Ind-2001e FMDVs showed that the 18 Myanmar isolates formed three distinct genetic groups (Figure 3). Two isolates from Yangon State in 2016, O/MYA/Yan/3/2016 and O/MYA/Yan/5/2016, were classified as group 1, which also included isolates from Central, South, Southeast, and East Asia during the same period. Two isolates from the Sagaing Region in 2019 were classified as group 2. Other isolates from 2019, 2020, and 2022 were classified as group 3. Group 3 included isolates that occurred in the southern, central, and northern parts of Myanmar and also included an isolate from Indonesia in 2022 (O/ISA/1/2022).

### 3.3. Comparison of the Pathogenicity With Field Isolates From 2016 to 2019 in Pigs via Intraoral Route

**Figure 4:**
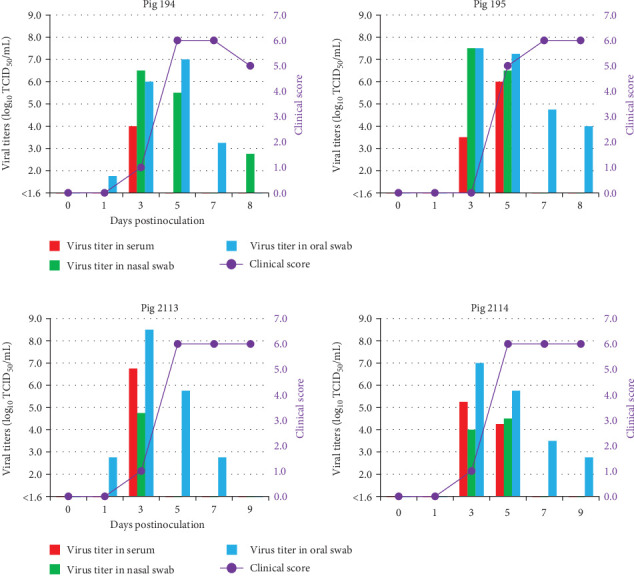
Viral titers of the clinical samples and clinical scores in pigs inoculated with field isolates in Myanmar via the intraoral route in Experiment 1. The *x*-axis shows the number of days postinoculation. The red, green, and blue bars indicate the viral titers (expressed as log_10_ 50% tissue culture infectious dose (TCID_50_)/mL) in the serum, nasal swab, and oral swab samples, respectively, on the left *y*-axis. The clinical scores are shown on the right *y*-axis. Pigs inoculated with (A) O/MYA/Yan/5/2016 and (B) O/MYA/Mgy/11/2019 strains.

In Experiment 1, the pathogenicity of the two strains, classified into separate genetic groups in the phylogenetic analysis of ORF, was clarified in pigs through the intraoral inoculation. Pigs inoculated with O/MYA/Yan/5/2016 (Nos. 194 and 195) showed vesicular lesions around the oral cavity and feet at 4 dpi (Table 2), reaching the maximum clinical score of 6 (Figure 4A). Representative lesions of each pig were presented in Supporting Information 1: Figure S1A. Viremia and virus excretion in nasal and oral swabs were observed between 1–8 dpi, with the maximum viral titers in serum, nasal, and oral swab samples reaching 10^6.0^, 10^7.5^, and 10^7.5^ TCID_50_/mL, respectively, as determined by virus titration using LFPK-α_v_β_6_ cell (Table 2). Viral RNAs were similarly detected in serum, oral, and nasal swab samples from 3 to 8 dpi, and antibodies were detected from 7 dpi (Table 3).

Pigs inoculated with O/MYA/Mgy/11/2019 (Nos. 2113, 2114) showed vesicular lesions around the oral cavity, snout, and feet from 3 to 5 dpi (Table 2), reaching the maximum clinical score of 6 (Figure 4B). Representative lesions of each pig were presented in Supporting Information 1: Figure S1B. Viremia and viral excretion in nasal and oral swabs were observed between 1 and 9 dpi, with the maximum viral titers in the serum, nasal, and oral swab samples reaching 10^6.8^, 10^4.8^, and 10^8.5^ TCID_50_/mL, respectively (Table 2). Viral RNAs were similarly detected in serum, oral, and nasal swab samples from 1 to 9 dpi, and antibodies were detected from 10 dpi (Table 3).

### 3.4. Pathogenicity With O/MYA/11/Mgy/2019 in Cattle and Pigs via Intradermal Route

**Figure 5:**
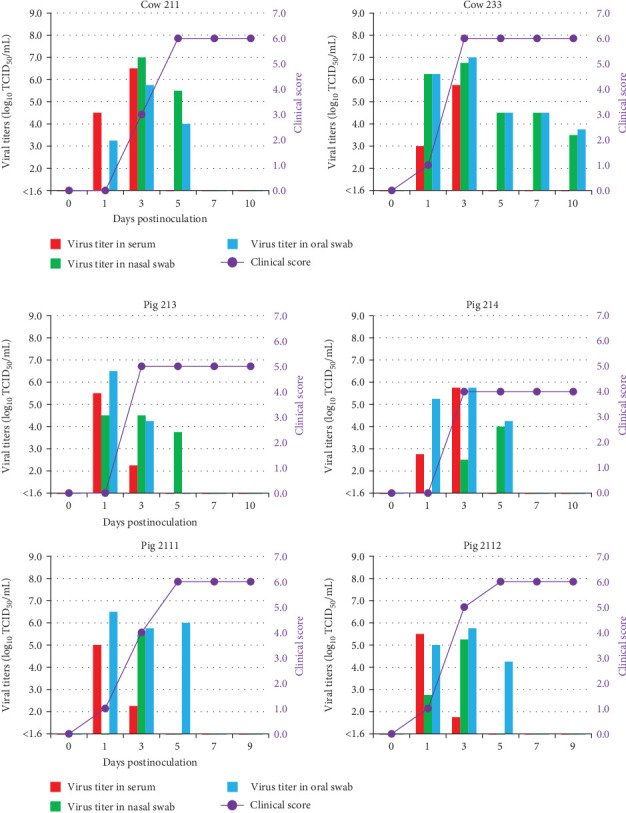
Viral titers of the clinical samples and clinical scores in animals inoculated with O/MYA/Mgy/11/2019 via the intradermal route in Experiment 2. The *x*-axis shows the number of days postinoculation. The red, green, and blue bars indicate viral titers (expressed as log_10_ 50% tissue culture infectious dose (TCID_50_)/mL) in the serum, nasal, and oral swab samples, respectively, on the left *y*-axis. The clinical scores are shown on the right *y*-axis. (A) Cattle inoculated with O/MYA/Mgy/11/2019. (B) Pigs inoculated with O/MYA/Mgy/11/2019.

In Experiment 2, the pathogenicity of O/MYA/11/Mgy/2019, classified into group 3, circulates widely in Myanmar, and closely related to O/ISA/1/2022 in the phylogenetic analysis of ORF, was clarified in cattle and pigs through the intradermal inoculation. Two cattle (Nos. 211 and 233) showed vesicular lesions around the oral cavity, snout, and feet at 2–4 dpi (Table 2), reaching the maximum clinical score of 6 (Figure 5A). Representative lesions of each cow were presented in Supporting Information 2: Figure S2A. Viremia and virus excretion in nasal and oral swabs were observed between 1–10 dpi, with the maximum viral titers in serum, nasal, and oral swab samples reaching 10^6.5^, 10^7.0^, and 10^7.0^ TCID_50_/mL, respectively (Table 2). Viral RNAs were similarly detected in serum, oral, and nasal swab samples from 1 to 10 dpi, and antibodies were detected from 7 dpi (Table 3).

Four pigs (Nos. 213, 214, 2111, and 2112) showed vesicular lesions around the oral cavity, snout, and feet at 1–3 dpi (Table 2), reaching the maximum clinical score of 6 (Figure 5B). Representative lesions of each pig were presented in Supporting Information 2: Figure S2B. Viremia and viral excretion in nasal and oral swabs were observed between 1 and 5 dpi, with the maximum viral titers in the serum, nasal, and oral swab samples reaching 10^5.8^, 10^5.5^, and 10^6.5^ TCID_50_/mL, respectively (Table 2). Viral RNAs were similarly detected in serum, oral, and nasal swab samples from 1 to 10 dpi and antibodies were detected from 5 to 10 dpi (Table 3).

Cattle and pigs inoculated with O/JPN/2010 [26] showed a similar period of vesicular development, viremia, and viral excretions as those observed in the present study (Supporting Information 4: Table S2 and Supporting Information 3: Figure S3).

## 4. Discussion

Phylogenetic characterization of circulating FMDVs and pathogenicity in susceptible animals are essential for establishing and renovating countermeasures against FMDV. In this study, the FMDVs detected from 2016 to 2022 were found in various regions, from Kachin State in the north to the Tanintharyi Region in the south (Figure 1 and Table 1). Phylogenetic analysis revealed that viruses detected from 2016 to 2019 in this study belonged to multiple serotypes and subtypes, including the ME-SA/Ind-2001e and SEA/Mya-98 for serotype O and ASIA/Sea-97 for serotype A. Additionally, a serotype Asia1 outbreak was reported in 2017 [14], indicating the presence of three distinct serotypes were present in Myanmar during this period. However, although the number of FMDVs in this study was limited, 19 FMDVs detected after 2019 were classified as the ME-SA/Ind-2001e (Figure 2). Comparison of VP1 sequences of serotype O FMDVs detected in Myanmar with those in other countries indicated that ME-SA/Ind-2001e isolates in Myanmar were closely related to isolates from Pools 1 and 2. Additionally, SEA/Mya-98 isolates were closely related to isolates from Pool 1. The epidemic situation within serotype O in Myanmar suggests a similar pattern as shown in reports from neighboring countries in Pool 1 such as Cambodia, the Lao People's Democratic Republic, Thailand, Vietnam, and China [31, 32].

Whole genome analysis of ME-SA/Ind-2001e FMDVs revealed that FMDVs detected in Myanmar after 2019 belong to different genetic groups than those isolated in 2016 (Figure 3). Although the sequence data were limited, the phylogenic analysis showed that the Myanmar isolates from 2016 were closely related to those from neighboring regions categorized in Pool 1. In contrast, the Myanmar isolates from 2019, 2020, and 2022 were closely related to O/ISA/1/2022. These viruses were identified across a wide geographic area in Myanmar. These findings confirm the phylogenetic relationship between FMDVs circulating in Myanmar and those in the surrounding Asian region, particularly those that have emerged in Southeast Asian countries in recent years, emphasizing the importance of surveillance in this country.

In Experiment 1, the pathogenicity of viral strains from different outbreaks and genetic groups of the ME-SA/Ind-2001e was compared in pigs. Pigs inoculated with O/MYA/Yan/5/2016 in group 1 and O/MYA/Mgy/11/2019 in group 3 showed similar patterns of vesicular development, viremia, and viral excretion patterns in viral titer or periods. Therefore, the pathogenicity of two field isolates in pigs is presumed to be comparable, despite being in separate genetic groups. In Experiment 2, O/MYA/Mgy/11/2019 exhibited typical pathogenicity in both cattle and pigs, comparable to that of O/JPN/2010, which caused severe outbreaks in Japan. Furthermore, viral excretion patterns in cattle and pigs infected with both O/MYA/11/Mgy/2019 and O/JPN/2010 isolates showed maximum viral titers in nasal and oral swabs were confirmed to be above 10^6.0^ TCID_50_/mL, exceeding the minimum infectious dose in pigs via the intraoral route previously reported for the two FMDV isolates [25, 33]. Therefore, if this viral strain enters FMD-free areas with a high density of naive livestock, particularly pig farms, it could cause a significant outbreak. Thus, early detection is crucial for implementing prevention and mitigating the spread of the Myanmar FMDVs, which exhibit typical pathogenicity. Our experimental infections demonstrated that existing diagnostic methods based on the WOAH Terrestrial Manual could effectively detect infectious viruses, genes, and antibodies in infected animals. Considering that FMDVs detected in neighboring countries are genetically closely related to isolates in this study, it is possible that existing methods are applicable for the diagnosis of viruses circulating in Myanmar and the surrounding Asian region. This highlights the need to continue diagnosing suspected cases and conducting surveillance using the available methods to prevent the uncontrolled spread of FMD.

## 5. Conclusions

We identified: (1) the phylogenetic characterization of circulating FMDV in Myanmar from 2016 to 2022, (2) the phylogenetic relationships between viruses in Myanmar and those emerging in neighboring Asian countries in recent years, and (3) the pathogenicity in cattle and pigs with typical clinical signs and virus excretion using existing diagnostic methods. FMDV surveillance and countermeasures, particularly in Myanmar, which plays a crucial role in the epidemic situation, are vital for preventing and eradicating outbreaks in Southeast and East Asia.

## Figures and Tables

**Table 1:** Overview of the virus isolates or clinical samples in Myanmar between 2016 and 2022.

Year	Name	Administrative divisions	Town	Samples	Species	GenBank accession no.
Virus isolates						

2016	O/MYA/Yan/3/2016^a^	Yangon	−	−	Cattle	LC438822
	**O/MYA/Yan/5/2016** ^**a**^	Yangon	−	−	Cattle	LC438823

2019	O/MYA/Sg/9/2019	Sagaing	Kanbalu	Hoof epithelium	Cattle	LC604882
	**O/MYA/Mgy/11/2019**	Magway	Salin	Tongue epithelium	Cattle	LC604883
	O/MYA/Sg/12/2019	Sagaing	Wuntho	Hoof epithelium	Cattle	LC604884
	O/MYA/Sg/15/2019	Sagaing	Khin-U	Tongue epithelium	Cattle	LC604885
	O/MYA/Sg/16/2019	Sagaing	Ye-U	Tongue epithelium	Cattle	LC604886
	O/MYA/Sg/17/2019	Sagaing	Wetlet	Tongue epithelium	Cattle	LC604887
	O/MYA/Mgy/20/2019	Magway	Aunglan	Tongue epithelium	Cattle	LC604888
	O/MYA/Sg/21/2019	Sagaing	Inndaw	Tongue epithelium	Cattle	LC604889
	A/MYA/Ygn/24/2019	Yangon	Tikegyi	Hoof epithelium	Cattle	LC604890

Clinical samples						

2018	MYA 1/2018	Mandalay	Nyaung-U	Tongue epithelium	Cattle	LC603291

2019	MYA 3/2019	Magway	Taungdwingyi	Hoof epithelium	Cattle	LC603292
	MYA 4/2019	Magway	Taungdwingyi	Hoof epithelium	Cattle	LC603293
	MYA 5/2019	Magway	Taungdwingyi	Tongue epithelium	Cattle	LC603294
	MYA 7/2019	Sagaing	Kanbalu	Hoof epithelium	Cattle	LC603295
	MYA 8/2019	Sagaing	Kanbalu	Hoof epithelium	Cattle	LC603296
	MYA 10/2019	Kachin	Myitkyinar	Tongue epithelium	Cattle	LC603297
	MYA 13/2019	Sagaing	Wuntho	Tongue epithelium	Cattle	LC603298
	MYA 14/2019	Magway	Magway	Tongue epithelium	Cattle	LC603299
	MYA 18/2019	Magway	Aunglan	Hoof epithelium	Cattle	LC603300
	MYA 19/2019	Magway	Aunglan	Hoof epithelium	Cattle	LC603301

2020	MYA 1/2020	Tanintharyi	Myeik	Tongue epithelium	Cattle	LC815045
	MYA 2/2020	Tanintharyi	Myeik	Hoof epithelium	Pig	LC815046

2022	MYA 4/2022	Yangon	Teik Kyi	Tongue epithelium	Cattle	LC815047
	MYA 5/2022	Yangon	Teik Kyi	Tongue epithelium	Cattle	LC815048
	MYA 6/2022	Yangon	Teik Kyi	Tongue epithelium	Cattle	LC815049
	MYA 8/2022	Bago	Bago	Hoof epithelium	Cattle	LC815050
	MYA 9/2022	Yangon	Mingalardon	Tongue epithelium	Cattle	LC815051
	MYA 10/2022	Yangon	Hmawbi	Tongue epithelium	Cattle	LC815052
	MYA 11/2022	Yangon	Hmawbi	Tongue epithelium	Cattle	LC815053
	MYA 13/2022	Sagaing	Myaung	Tongue epithelium	Cattle	LC815054
	MYA 14/2022	Sagaing	Myaung	Tongue epithelium	Cattle	LC815055
	MYA 15/2022	Mandalay	Patheingyi	Tongue epithelium	Cattle	LC815056

*Note:* “−” Indicates lack of recorded data. Bold indicates virus strain used in experimental infection.

^a^Genome sequence data were previously reported [23].

**Table 2:** Detection period and peak clinical scores or viral titers observed in each group during experimental period.

Virus strain animal species	Detection period/peak clinical scores or viral titers			
	Clinical signs	Sera	Nasal swabs	Oral swabs
Experiment 1 (intraoral route)				
O/MYA/Yan/5/2016 pigs	4^a^/6^b^	3–5^c^/10^6.0d^	3–8^e^/10^7.5d^	1–8^e^/10^7.5d^
O/MYA/Mgy/11/2019 pigs	3–5/6	3–5/10^6.8^	3–5/10^4.8^	1–9/10^8.5^

Experiment 2 (intradermal route)				
O/MYA/Mgy/11/2019 cattle	2–4/6	1–3/10^6.5^	1–10/10^7.0^	1–10/10^7.0^
O/MYA/Mgy/11/2019 pigs	1–3/6	1–3/10^5.8^	1–5/10^5.5^	1–5/10^6.5^

^a^First observation day (days postinoculation: dpi) of vesicular development.

^b^Confirmed peak clinical scores.

^c^Confirmed period (dpi) of viremia.

^d^Peak viral titers (50% tissue culture infectious dose/mL) measured in sera, nasal, or oral swabs.

^e^Confirmed period (dpi) of virus excretion in nasal and oral swabs.

**Table 3:** Time-dependent detection of FMDV-specific genes from clinical samples by RT-PCR and antibody against serotype O FMDV structual protein by ELISA.

Animal number	Clinical samples or assay	Days postinoculation (dpi)					
		0	1	3	5	7	8/9/10^a^
Experiment 1 (intraoral route)							

O/MYA/Yan/5/2016 in pigs							

194	Serum	−	−	+	+	−	−
	Oral swab	−	−	+	+	+	+
	Nasal swab	−	−	+	+	+	+
	ELISA	−	−	−	−	+	+

195	Serum	−	−	+	+	−	−
	Oral swab	−	−	+	+	+	+
	Nasal swab	−	−	+	+	+	+
	ELISA	−	−	−	−	+	+

O/MYA/Mgy/11/2019 in pigs							

2113	Serum	−	−	+	+	−	−
	Oral swab	−	+	+	+	+	+
	Nasal swab	−	−	+	+	+	−
	ELISA	−	−	−	−	−	+

2114	Serum	−	−	+	+	−	−
	Oral swab	−	−	+	+	+	+
	Nasal swab	−	−	+	+	+	−
	ELISA	−	−	−	−	−	+

Experiment 2 (intradermal route)							

O/MYA/Mgy/11/2019 in cattle							

211	Serum	−	+	+	+	+	+
	Oral swab	−	+	+	+	+	+
	Nasal swab	−	+	+	+	+	+
	ELISA	−	−	−	−	+	+

233	Serum	−	+	+	+	−	−
	Oral swab	−	+	+	+	+	+
	Nasal swab	−	+	+	+	+	+
	ELISA	−	−	−	−	+	+

O/MYA/Mgy/11/2019 in pigs							

213	Serum	−	+	+	+	−	−
	Oral swab	−	+	+	+	+	+
	Nasal swab	−	+	+	+	+	+
	ELISA	−	−	−	+	+	+

214	Serum	−	+	+	+	−	−
	Oral swab	−	+	+	+	+	+
	Nasal swab	−	+	+	+	+	−
	ELISA	−	−	−	−	−	+

2111	Serum	−	+	+	−	−	−
	Oral swab	−	+	+	+	+	+
	Nasal swab	−	−	+	+	−	−
	ELISA	−	−	−	+	+	+

2112	Serum	−	+	+	−	−	−
	Oral swab	−	+	+	+	+	+
	Nasal swab	−	+	+	+	−	−
	ELISA	−	−	−	+	+	+

*Note:* “−” negative results. “+” positive results, indicated with grey shading.

^a^The clinical samples were collected from pigs 194 and 195 at 8 dpi; pigs 2113, 2114, 2111, and 2112 at 9 dpi; cows 211 and 233 as well as pigs 213 and 214 at 10 dpi.

## Data Availability

The data supporting the findings of this study are available from the corresponding authors upon reasonable request. Accession numbers of the viral sequences are listed in Table 1.

## Supporting Information

Supporting Information 1 Figure S1: Representative lesions observed in pigs after inoculation with field isolates from Myanmar in Experiment 1. The figure shows the representative lesions observed in pigs during Experiment 1. The red arrows indicate vesicles at predilection sites. (A) Pigs inoculated with O/MYA/Yan/5/2016, a: Lip of Pig 194 at 5 days postinoculation (dpi), b: Forelimbs of Pig 194 at 5 dpi, c: Tongue of Pig 195 at 7 dpi, d: Hindlimbs of Pig 195 at 7 dpi. (B) Pigs inoculated with O/MYA/Mgy/11/2019, e: Lip of Pig 2113 at 5 dpi, f: Forelimbs of Pig 2113 at 7 dpi, g: Lip of Pig 2114 at 5 dpi, h: Hindlimbs of Pig 2114 at 5 dpi.

Supporting Information 2 Figure S2: Representative lesions observed in cattle and pigs after inoculation with O/MYA/Mgy/11/2019 in Experiment 2. The figure shows the representative lesions observed in cattle and pigs during Experiment 2. The red arrows indicate vesicles at predilection sites. (A) Cattle inoculated with O/MYA/Mgy/11/2019, a: Nose of Cow 211 at 5 days postinoculation (dpi), b: Right forelimb of Cow 211 at 5 dpi, c: Lip of Cow 233 at 3 dpi, d: Left hindlimb of Cow 233 at 5 dpi. (B) Pigs inoculated with O/MYA/Mgy/11/2019, e: Lip of Pig 213 at 3 dpi, f: Hindlimbs of Pig 213 at 5 dpi, g: Hindlimbs of Pig 214 at 5 dpi, h: Lip of Pig 2111 at 5 dpi, i: Hindlimbs of Pig 2111 at 5 dpi, j: Lip of Pig 2112 at 5 dpi, k: Forelimbs of Pig 2112 at 7 dpi.

Supporting Information 3 Figure S3: Viral titers of clinical samples and clinical scores of cattle and pigs inoculated with O/JPN/2010. The figure shows viral titers in sera, nasal, and oral swab samples, and development of clinical scores in each cattle and pigs inoculated with O/JPN/2010 over the 7-days postinoculation. The *x*-axis shows the number of days postinoculation. The red, green, and blue bars indicate viral titers (expressed as log10 50% tissue culture infectious dose (TCID50)/mL) in the serum, nasal, and oral swab samples, respectively, on the left *y*-axis. The clinical scores are shown on the right *y*-axis. (A) Cattle inoculated with O/MYA/Mgy/11/2019. (B) Pigs inoculated with O/MYA/Mgy/11/2019. (A) Cattle inoculated with O/JPN/2010 and (B) pigs inoculated with O/JPN/2010.

Supporting Information 4 Table S1: Overview of the virus isolates used in experimental infection classified Serotype O, ME-SA topotype, Ind-2001e lineage. The table shows the information regarding the outbreak, passage history, and GenBank accession numbers of the virus isolates used in experimental infections. Table S2: Detection period and peak clinical scores or viral titers observed in cattle and pigs inoculated with O/JPN/2010. The table shows the first observation day of vesicular development, the peak of clinical scores, the duration of confirmed viremia and virus excretion in nasal and oral swabs, and the peak viral titers measured in serum, nasal, or oral swabs in cattle and pigs inoculated with O/JPN/2010.
