# Supplementary material for: Phylogenetic Characterization and Pathogenicity in Cattle and Pigs of Foot-and-Mouth Disease Viruses Circulating in Myanmar Between 2016 and 2022
Source: Transbound Emerg Dis. 2025 Oct 29;2025:1532487. doi: 10.1155/tbed/1532487 (PMC12588753; doi:10.1155/tbed/1532487)
Supplement: Supporting Information 1 — Figure S1: Representative lesions observed in pigs after inoculation with field isolates from Myanmar in Experiment 1. The figure shows the representative lesions observed in pigs during Experiment 1. The red arrows indicate vesicles at predilection sites. (A) Pigs inoculated with O/MYA/Yan/5/2016, a: Lip of Pig 194 at 5 days postinoculation (dpi), b: Forelimbs of Pig 194 at 5 dpi, c: Tongue of Pig 195 at 7 dpi, d: Hindlimbs of Pig 195 at 7 dpi. (B) Pigs inoculated with O/MYA/Mgy/11/2019, e: Lip of Pig 2113 at 5 dpi, f: Forelimbs of Pig 2113 at 7 dpi, g: Lip of Pig 2114 at 5 dpi, h: Hindlimbs of Pig 2114 at 5 dpi. [file 1532487.f1.pptx]

## Slide 1
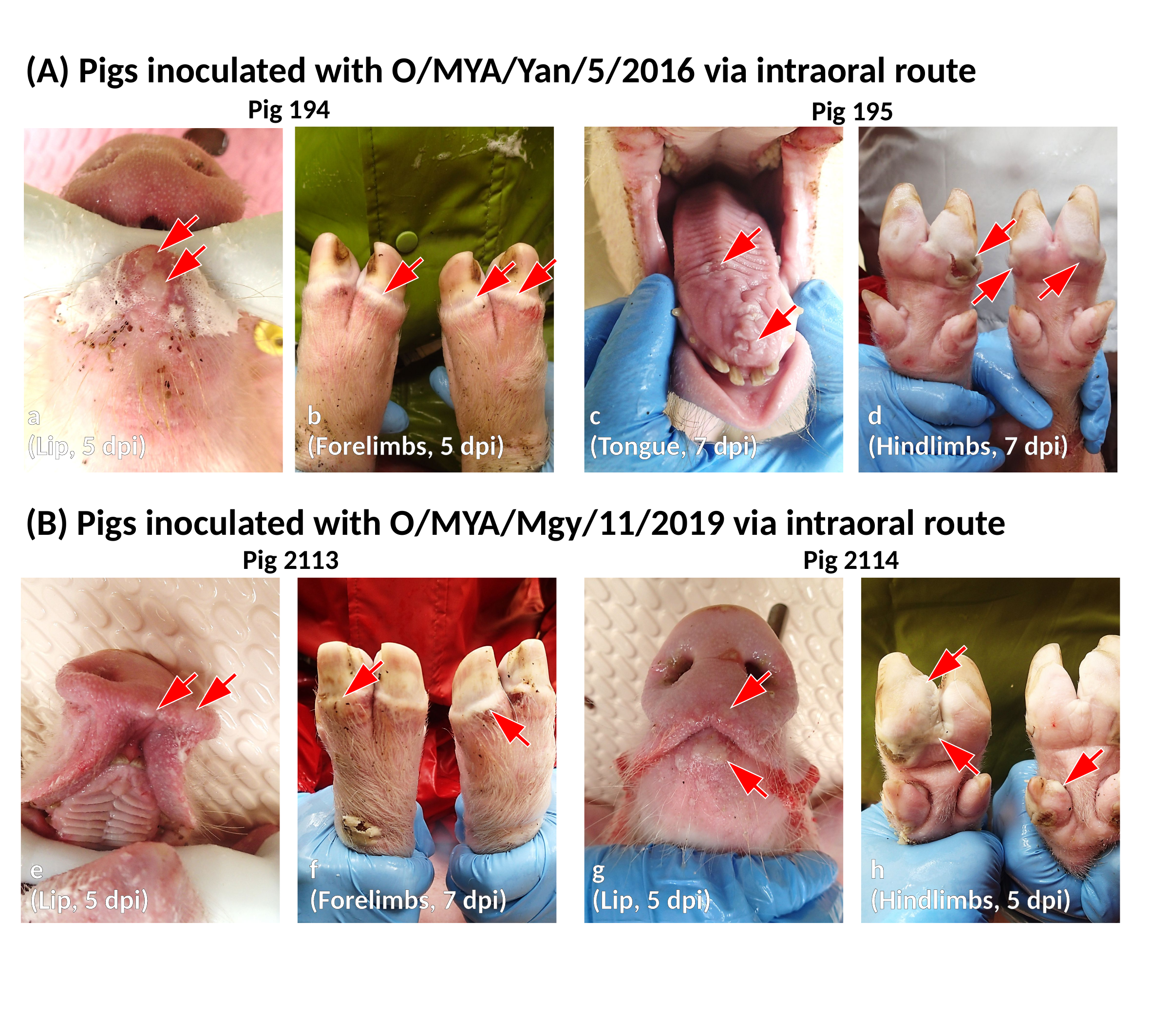

(A) Pigs inoculated with O/MYA/Yan/5/2016 via intraoral route
Pig 194
Pig 195
a
(Lip, 5 dpi)
c
(Tongue, 7 dpi)
d
(Hindlimbs, 7 dpi)
b
(Forelimbs, 5 dpi)
(B) Pigs inoculated with O/MYA/Mgy/11/2019 via intraoral route
Pig 2113
Pig 2114
e
(Lip, 5 dpi)
f
(Forelimbs, 7 dpi)
g
(Lip, 5 dpi)
h
(Hindlimbs, 5 dpi)
