# Supplementary material for: Phylogenetic Characterization and Pathogenicity in Cattle and Pigs of Foot-and-Mouth Disease Viruses Circulating in Myanmar Between 2016 and 2022
Source: Transbound Emerg Dis. 2025 Oct 29;2025:1532487. doi: 10.1155/tbed/1532487 (PMC12588753; doi:10.1155/tbed/1532487)
Supplement: Supporting Information 3 — Figure S3: Viral titers of clinical samples and clinical scores of cattle and pigs inoculated with O/JPN/2010. The figure shows viral titers in sera, nasal, and oral swab samples, and development of clinical scores in each cattle and pigs inoculated with O/JPN/2010 over the 7-days postinoculation. The x-axis shows the number of days postinoculation. The red, green, and blue bars indicate viral titers (expressed as log10 50% tissue culture infectious dose (TCID50)/mL) in the serum, nasal, and oral swab samples, respectively, on the left y-axis. The clinical scores are shown on the right y-axis. (A) Cattle inoculated with O/MYA/Mgy/11/2019. (B) Pigs inoculated with O/MYA/Mgy/11/2019. (A) Cattle inoculated with O/JPN/2010 and (B) pigs inoculated with O/JPN/2010. [file 1532487.f3.pptx]

## Slide 1
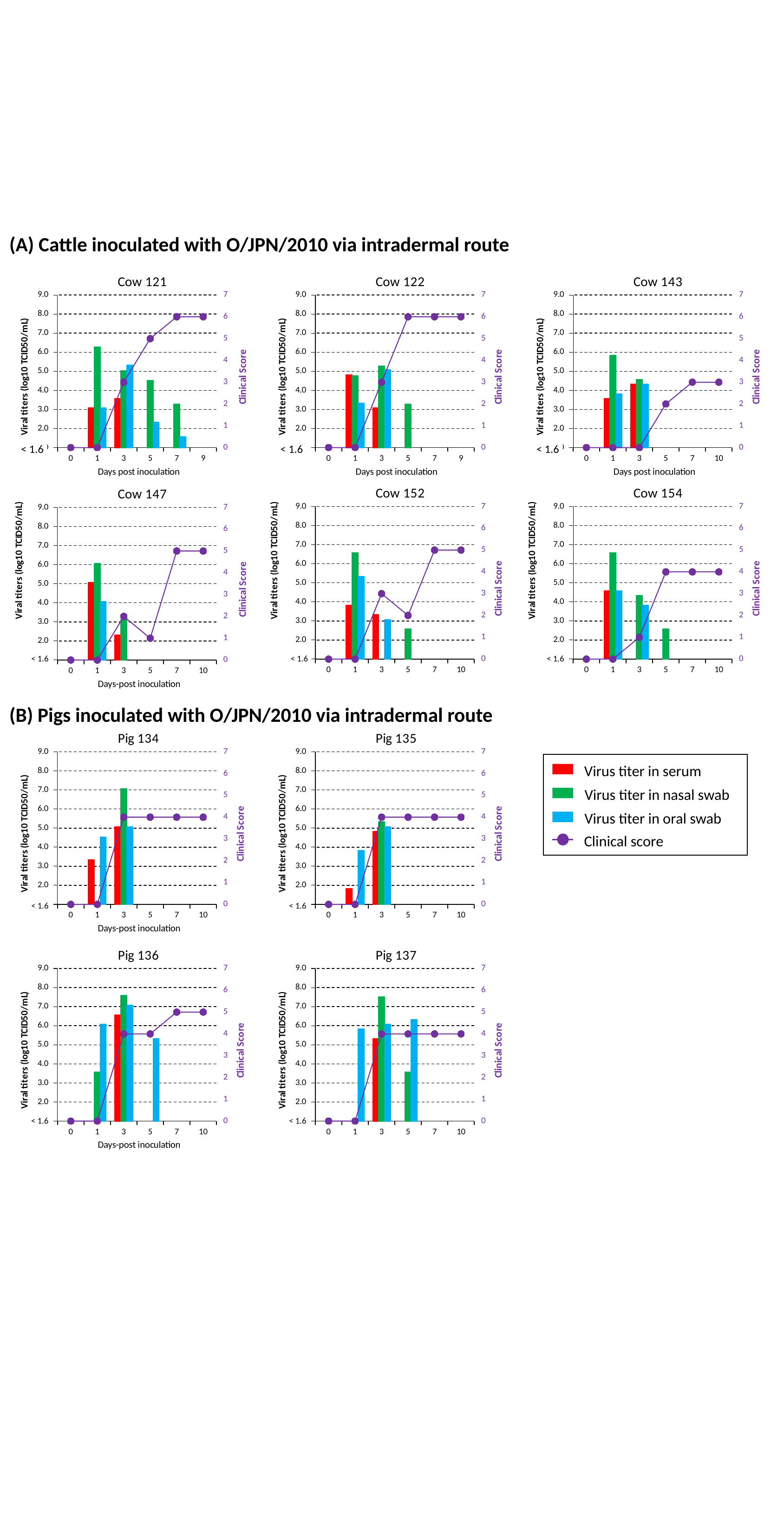

(A) Cattle inoculated with O/JPN/2010 via intradermal route
### Chart: Cow 121
| Category | | | | |
|---|---|---|---|---|
| 0 | 0.0 | 0.0 | 0.0 | 0.0 |
| 1 | 3.073908740944319 | 6.272878745280337 | 3.073908740944319 | 0.0 |
| 3 | 3.573908740944319 | 5.022878745280337 | 5.3239087409443195 | 3.0 |
| 5 | 0.0 | 4.522878745280337 | 2.323908740944319 | 5.0 |
| 7 | 0.0 | 3.2728787452803374 | 1.5739087409443189 | 6.0 |
| 9 | 0.0 | 0.0 | 0.0 | 6.0 |
### Chart: Cow 122
| Category | | | | |
|---|---|---|---|---|
| 0 | 0.0 | 0.0 | 0.0 | 0.0 |
| 1 | 4.823908740944319 | 4.772878745280338 | 3.323908740944319 | 0.0 |
| 3 | 3.073908740944319 | 5.272878745280338 | 5.073908740944319 | 3.0 |
| 5 | 0.0 | 3.2728787452803374 | 0.0 | 6.0 |
| 7 | 0.0 | 0.0 | 0.0 | 6.0 |
| 9 | 0.0 | 0.0 | 0.0 | 6.0 |
### Chart: Cow 143
| Category | | | | |
|---|---|---|---|---|
| 0 | 0.0 | 0.0 | 0.0 | 0.0 |
| 1 | 3.573908740944319 | 5.823908740944319 | 3.8239087409443187 | 0.0 |
| 3 | 4.323908740944319 | 4.5739087409443195 | 4.323908740944319 | 0.0 |
| 5 | 0.0 | 0.0 | 0.0 | 2.0 |
| 7 | 0.0 | 0.0 | 0.0 | 3.0 |
| 10 | 0.0 | 0.0 | 0.0 | 3.0 |< 1.6
< 1.6
< 1.6
### Chart: Cow 152
| Category | | | | |
|---|---|---|---|---|
| 0 | 0.0 | 0.0 | 0.0 | 0.0 |
| 1 | 3.8239087409443187 | 6.5739087409443195 | 5.3239087409443195 | 0.0 |
| 3 | 3.323908740944319 | 0.0 | 3.073908740944319 | 3.0 |
| 5 | 0.0 | 2.5739087409443187 | 0.0 | 2.0 |
| 7 | 0.0 | 0.0 | 0.0 | 5.0 |
| 10 | 0.0 | 0.0 | 0.0 | 5.0 |
### Chart: Cow 154
| Category | | | | |
|---|---|---|---|---|
| 0 | 0.0 | 0.0 | 0.0 | 0.0 |
| 1 | 4.5739087409443195 | 6.5739087409443195 | 4.5739087409443195 | 0.0 |
| 3 | 0.0 | 4.323908740944319 | 3.8239087409443187 | 1.0 |
| 5 | 0.0 | 2.5739087409443187 | 0.0 | 4.0 |
| 7 | 0.0 | 0.0 | 0.0 | 4.0 |
| 10 | 0.0 | 0.0 | 0.0 | 4.0 |
### Chart: Cow 147
| Category | | | | |
|---|---|---|---|---|
| 0 | 0.0 | 0.0 | 0.0 | 0.0 |
| 1 | 5.073908740944319 | 6.0739087409443195 | 4.0739087409443195 | 0.0 |
| 3 | 2.323908740944319 | 3.1249387366083 | 0.0 | 2.0 |
| 5 | 0.0 | 0.0 | 0.0 | 1.0 |
| 7 | 0.0 | 0.0 | 0.0 | 5.0 |
| 10 | 0.0 | 0.0 | 0.0 | 5.0 |< 1.6
< 1.6
< 1.6
(B) Pigs inoculated with O/JPN/2010 via intradermal route
### Chart: Pig 134
| Category | | | | |
|---|---|---|---|---|
| 0 | 0.0 | 0.0 | 0.0 | 0.0 |
| 1 | 3.323908740944319 | None | 4.522878745280337 | 0.0 |
| 3 | 5.073908740944319 | 7.073908740944319 | 5.073908740944319 | 4.0 |
| 5 | None | None | None | 4.0 |
| 7 | 0.0 | 0.0 | 0.0 | 4.0 |
| 10 | 0.0 | 0.0 | 0.0 | 4.0 |
### Chart: Pig 135
| Category | | | | |
|---|---|---|---|---|
| 0 | 0.0 | 0.0 | 0.0 | 0.0 |
| 1 | 1.8239087409443189 | None | 3.8239087409443187 | 0.0 |
| 3 | 4.823908740944319 | 5.3239087409443195 | 5.073908740944319 | 4.0 |
| 5 | None | None | None | 4.0 |
| 7 | None | None | None | 4.0 |
| 10 | None | None | None | 4.0 |
Virus titer in serum
Virus titer in nasal swab
Virus titer in oral swab
Clinical score
< 1.6
< 1.6
### Chart: Pig 136
| Category | | | | |
|---|---|---|---|---|
| 0 | 0.0 | 0.0 | 0.0 | 0.0 |
| 1 | None | 3.573908740944319 | 6.0739087409443195 | 0.0 |
| 3 | 6.5739087409443195 | 7.5739087409443195 | 7.073908740944319 | 4.0 |
| 5 | None | None | 5.3239087409443195 | 4.0 |
| 7 | None | None | None | 5.0 |
| 10 | None | None | None | 5.0 |
### Chart: Pig 137
| Category | | | | |
|---|---|---|---|---|
| 0 | 0.0 | 0.0 | 0.0 | 0.0 |
| 1 | None | None | 5.823908740944319 | 0.0 |
| 3 | 5.3239087409443195 | 7.522878745280337 | 6.0739087409443195 | 4.0 |
| 5 | None | 3.573908740944319 | 6.323908740944319 | 4.0 |
| 7 | None | None | None | 4.0 |
| 10 | None | None | None | 4.0 |< 1.6
< 1.6
